# Deep action learning enables robust 3D segmentation of body organs in various CT and MRI images

**DOI:** 10.1038/s41598-021-82370-6

**Published:** 2021-02-08

**Authors:** Xia Zhong, Mario Amrehn, Nishant Ravikumar, Shuqing Chen, Norbert Strobel, Annette Birkhold, Markus Kowarschik, Rebecca Fahrig, Andreas Maier

**Affiliations:** 1 Pattern Recognition Lab, Friedrich-Alexander University, Erlangen-Nürnberg, Germany; 2 Institute of Medical Engineering, University of Applied Sciences, Würzburg-Schweinfurt, Germany; 3 Siemens Healthcare GmbH, Forchheim, Germany

**Keywords:** Computer science, Medical imaging

## Abstract

In this study, we propose a novel point cloud based 3D registration and segmentation framework using reinforcement learning. An artificial agent, implemented as a distinct actor based on value networks, is trained to predict the optimal piece-wise linear transformation of a point cloud for the joint tasks of registration and segmentation. The actor network estimates a set of plausible actions and the value network aims to select the optimal action for the current observation. Point-wise features that comprise spatial positions (and surface normal vectors in the case of structured meshes), and their corresponding image features, are used to encode the observation and represent the underlying 3D volume. The actor and value networks are applied iteratively to estimate a sequence of transformations that enable accurate delineation of object boundaries. The proposed approach was extensively evaluated in both segmentation and registration tasks using a variety of challenging clinical datasets. Our method has fewer trainable parameters and lower computational complexity compared to the 3D U-Net, and it is independent of the volume resolution. We show that the proposed method is applicable to mono- and multi-modal segmentation tasks, achieving significant improvements over the state-of-the-art for the latter. The flexibility of the proposed framework is further demonstrated for a multi-modal registration application. As we learn to predict actions rather than a target, the proposed method is more robust compared to the 3D U-Net when dealing with previously unseen datasets, acquired using different protocols or modalities. As a result, the proposed method provides a promising multi-purpose segmentation and registration framework, particular in the context of image-guided interventions.

## Introduction

Segmentation and registration of anatomical structures in 3D imaging are important pre-processing steps for clinical applications such as computer aided-diagnosis, treatment planning, or X-ray dose management. Developing such segmentation and registration method for practical use is challenging, as the method has to be robust w.r.t. patient anatomy, scan protocols, as well as type, amount, and injection rate of contrast agents. Additionally, any algorithm, in particular when used in an intra-procedural setting, should involve minimum user interaction and must have a fast run-time. While many approaches exist treating segmentation and registration as separate tasks, an approach solving both problems simultaneously as proposed in^1^ is therefore very appealing. These methods are usually referred to as model-based or atlas-based segmentation approaches. Employing such a framework makes it possible to align pre-operatively segmented datasets with intra-operative or post-operative data, and segment the latter. Despite these advantages, e.g., a good segmentation performance, in particular when using neural networks, during inference the computational complexity and run-time of such techniques is quite high. Reducing the inference time in multi-modal segmentation and registration is, however, essential to enable applications in interventional imaging and endovascular treatment settings, settings such as liver embolization or transarterial chemo embolization (TRACE). To address this challenge, we introduce a general scheme for joint point cloud-based joint segmentation and registration based on reinforcement learning.

### 3D image segmentation

Image segmentation techniques can be categorized and formulated in different ways. In this article we categorize the method into model-based (boundary seeking), image-based (voxel-wise classification) and hybrid methods. Model-based methods deform the current estimate of an object’s boundary to either better conform to a learned appearance, or to obtain an improved alignment with a set of features designed to represent the structure of interest. This is usually obtained by minimizing a suitable energy function. Object boundaries may be represented in parametric or non-parametric form. They may be updated: (1) directly in 3D space using an active shape model (ASM)^2^, an active appearance model (AAM)^3^, or a sparse shape model^4^; (2) by altering its representation in 4D hyperspace using level-sets^5^, or (3) by deforming a template, e.g., by fitting a probabilistic atlas to the volume of interest^6^. In image-based methods, each voxel in the volume is segmented. This group of techniques comprises basic thresholding and Graph-cut^7,8^. In addition, there is unsupervised clustering^9^ and artificial neural networks^10,11^. Hybrid methods are a combination of model-based and image-based approaches^12^.

With the introduction of convolutional neural networks (CNNs) in recent years, image segmentation approaches have improved substantially. CNNs have been applied to both image-based and model-based segmentation problems outperforming traditional techniques. With regards to the former, the most noticeable breakthrough was the introduction of the U-Net for 2D segmentation by Ronneberger et al. ^10^ Subsequently, different variations of the U-Net were proposed, which have extended the method to 3D^13,14^, dealt with the issue of class imbalance by adapting the loss function appropriately^15^, introduced residual blocks^16^, incorporated adversarial learning^17^, or introduced attention mechanisms into the network^18^. For model-based settings, CNNs have been used to predict the deformation of a segmented template^19^, enabling single- and multi-atlas based segmentation^20^. Although these methods have shown great success and found widespread adoption in most medical image segmentation tasks, there are some disadvantages. For example, 3D-CNNs are computationally expensive with a complexity of ${\mathcal {O}}(n^3)$ during inference where *n* denotes the volume size. Although efforts have been made to accelerate the U-Net for 3D, the segmentation of a volume in its original resolution still needs about $100\,$s using an NVIDIA Titan X GPU^14^. Additionally, it is difficult for CNN-based approaches to impose geometric constraints using a single network. Some recent work uses graph networks^21^ or CNN for surface segmentation^22^ to tackle this problem. Often post-processing steps are necessary to enforce connectivity and to ensure boundary smoothness. Finally, CNNs are rather sensitive to the dataset. Fine-tuning or transfer learning is usually required in order to apply a pre-trained CNN to a new dataset, especially when the data is acquired differently relative to the data used for pre-training^23^. The difference may, e.g., be due to the use of a different scan protocol, e.g., a change in tube voltage or a different reconstruction matrix size when using computed tomography (CT). It could also be caused by use of an entirely different imaging modality, for example, magnetic resonance tomography (MRT) instead of CT. These factors inhibit the application of conventional CNN-based segmentation approaches (such as the U-Net) in clinical settings, where different imaging devices from various vendors set at site-specific acquisition protocols are used.

Parallel to this branch of deep learning approaches, reinforcement learning (RL) has also been investigated for image analysis. For example Krebs et al.^24^ used RL to estimate the modes of a 3D deformation field for a volume. Here, the modes, which are likely to increase the final segmentation accuracy, are iteratively estimated by the agent. Mo et al. ^25^ applied RL to train an agent for locating the boundary points of objects in 2D images. The agent successively identifies and adds boundary locations at each step and, on convergence, the final enclosure of the trajectory is considered as the segmentation result.

### Reinforcement learning in medical image processing

In recent years, there have been significant improvements in the capabilities of RL-based systems. With the introduction of deep *Q*-networks (DQN)^26^ separating policy and value networks^27^, and actor-critic networks^28^, RL has shown great success in autonomously acting in simulated constrained environments, e.g., chess and video games. Furthermore, there is research showing that RL also has great potential in medical image processing, e.g., in anatomical landmark detection^29,30^, rigid image registration^31^, and non-rigid image registration^24^. A comprehensive review of RL is out of the scope of this paper, however, we introduce the basic concepts of RL relevant to this work, and adaptation.

#### Finite Markov decision processes

The fomulation of RL is closely related to the Finite Markov Decision Process (MDP). The Finite Markov Decision Process comprises a set of possible states ${\varvec{s}}_t \in {\mathcal {S}}_t$, and a set of possible actions ${\varvec{a}}_t \in {\mathcal {A}}_t$ at time step *t*. Here, ${\mathcal {S}}_t$ denotes the set of all possible states, while ${\mathcal {A}}_t$ refers to the set of all actions. In addition, for each action ${\varvec{a}}_t$, we have an associated probability $\pi _t({\varvec{a}}_t|{\varvec{s}}_t)$ and an associated immediate reward $r_t({\varvec{a}}_t|{\varvec{s}}_t) \in {{\mathcal {R}}_{t}}$ with ${\mathcal {R}}_{t}$ denoting the set of rewards for all possible actions ${\mathcal {A}}_t$. At each time step *t*, the environment updates the state ${\varvec{s}}_t$. This yields a new immediate reward $r_{t}$ for each action ${\varvec{a}}_t \in {\mathcal {A}}_t$. The goal of RL is to train this artificial agent such that it learns an optimal policy $\pi ^*$ for maximizing the expected cumulative reward $r_t$ over time step *t*. An artificial agent determines in each step the optimal action ${\varvec{a}}_t^* \in {\mathcal {A}}_t$ in state ${\varvec{s}}_t$ in time step *t* by selecting the action with the highest probability $\pi _t{({\varvec{a}}_t|{\varvec{s}}_t)}$. In other words, future rewards are taken into account when deciding on an optimal action ${\varvec{a}}_t^*$. This can be expressed using the optimal action-value (*Q*-value) function,

1 $$\begin{aligned} Q^*({\varvec{s}},{\varvec{a}}) = \max _{\pi _t}{\mathbb {E}}\left[ \sum _{\tau =t}^{\infty }\gamma ^{\tau -t} r_t|{\varvec{s}} = {\varvec{s}}_t, {\varvec{a}} = {\varvec{a}}_t, \pi _t\right] \end{aligned}$$

In this equation, the scalar $\gamma \in (0,1]$ denotes the discount factor. As the name suggests, in finite MDP the sets of possible actions ${\mathcal {A}}_t$ and states ${\mathcal {S}}_t$ are finite. We use the symbol $^*$ to denote the optimal value of a given variable. An artificial agent with hyperparameters $\theta$ is trained to approximate the $Q^*$-value, by selecting actions ${\varvec{a}}_t^*$ associated with the highest *Q*-value, at each time step *t*. This is referred to as *Q*-learning, and can be formulated as,

2 $$\begin{aligned} \theta ^*= & {} \text {arg}\min _{\theta } \Vert r_t + \gamma \max _{{\varvec{a}}_{t+1}}Q({\varvec{s}}_{t+1}, {\varvec{a}}_{t+1};\theta ) - Q({\varvec{s}}_t,{\varvec{a}}_t;\theta )\Vert _2^2 \end{aligned}$$

3 $$\begin{aligned} {\varvec{a}}_t^*= & {} \text {arg}\max _{{\varvec{a}}_t \in {{\mathcal {A}}_t}}Q({\varvec{s}}_t,{\varvec{a}}_t;\theta ) \end{aligned}$$

As the goal of the reinforcement learning is to find ${\varvec{a}}^*_t$ for each time step *t*, the agent effectively predicts the trajectory of the actions undertaken. Such an optimization problem is highly non-convex, as different paths could lead to the same destination.

#### Continuous action space

When applying RL in medical image processing tasks, one of the major challenges is the action space. In finite MDP, the possible actions in any step belongs the discrete finite set ${\mathcal {A}}_t$. This is however not the case for most image processing tasks. In case of a 3D rigid registration application, the possible actions, e.g., rigid transformations in any given state ${\varvec{a}}_t \in {\mathbb {R}}^6$ is in a continuous space.

To deal with a continuous action space, we can either convert the problem to a finite MDP, or work in the continuous action space directly. Discretization^29,31^ or dimensionality reduction^24^ can be used to construct a finite action set, thereby converting the problem to a finite MDP. Here, the policy is learned using the deep *Q*-learning approach. The agent predicts the best action ${\varvec{a}}_t \in {{\mathcal {A}}_t}$ given the current state ${\varvec{s}}_t$. Alternatively, we could work with the continuous action space directly. To this end, the agent would need to be implemented as a parameterized actor. The agent would then estimate the action directly for each given state^25,32,33^.

#### State and observation

Another important difference to finite MDP is that the state in medical imaging applications is defined by the underlying 2D images or 3D volumes. In the implementation of an agent, however, the extracted ROIs in the 2D images or 3D volume are considered the current state. Strictly speaking, it is a partial observation ${\varvec{o}}_t$ of the state. The difference between the state and its observation does not change the general pipeline, as deep RL enables networks to be trained using states or observations. The partial observation effect, however, imposes implicit restrictions on both the optimal policy $\pi ^*$ as well as action ${\varvec{a}}_t^*$ possible for any given observation ${\varvec{o}}_t$. The agent can in theory only estimate a sub-optimal policy $\pi _t$ or action ${\varvec{a}}_t$ due to missing information. This may be addressed by designing a suitable multi-scale strategy^30^, wherein, the RL algorithm is formulated to estimate the scale level of the observation.

### Contribution

The main contribution of this paper is a novel point cloud based registration and segmentation method using reinforcement learning. An artificial agent is trained to predict a sequence of actions (in our case transformations) to align a point cloud *estimate* to the *ground truth*, given the current state. The agent is implemented as an *actor*-*value* network, where the *actor* estimates possible actions, such as affine or non-rigid transformations for the point cloud, as a finite set ${\mathcal {A}}_t$. The *value* network, on the other hand, determines the best action. At each time step, the agent predicts both the action and the associated expected action value (*Q*-value) based on the observed current state. The proposed approach offers several advantages over existing segmentation techniques, such as: (1) compared to conventional model-based segmentation methods, the proposed method does not require a computationally expensive energy function to guide and predict the deformation of the point could (or vertices) in the test phase; (2) in contrast to the CNN based methods, the proposed approach uses partial observations instead of the complete state to estimate the deformation. This contributes to a significant reduction in computational complexity; (3) unlike existing RL-based registration techniques^24,25^, we estimate the action directly for the whole point cloud in a continuous space, and we do not require a predefined deformation model. The separation of the *actor* and *value* networks facilitates the estimation of the trajectory and helps to incorporate the future reward. Unlike actor-critic methods, where the actions are taken from a predefined finite set and where the statistical distribution of the policy is learned during the training process, the proposed method predicts actions from a continues space and determines the policy using *Q*-learning.

## Method

We refer to the current distribution of vertices at time step *t* placed inside a 3D volume as *estimates*, denoted by ${\varvec{V}}_t \in {\mathbb {R}}^{N \times 3}$, where *N* denotes the number of vertices. The matrix ${\varvec{G}} \in {\mathbb {R}}^{M \times 3}$ , referred to as the *ground truth*, denotes the corresponding target distribution with *M* vertices. The goal of a model-based segmentation or registration algorithm is to find a transformation function ${\mathcal {T}}$ such that the distance between the transformed *estimates* and the *ground truth* is minimized. The function ${\text {dist}}(\cdot , \cdot )$ calculates the average symmetric distance between two point clouds.

4 $$\begin{aligned} {\mathcal {T}}^*= \text {arg}\min _{\mathcal {T}} \left( {\text {dist}}({\mathcal {T}}({{\varvec{V}}_t}), {\varvec{G}})\right) \end{aligned}$$

We can reformulate this minimization problem within a reinforcement learning framework as an infinite MDP, where the immediate reward $r_t$ denotes the decrease in distance between the *estimates* ${\varvec{V}}_t$ and the *ground truth* ${\varvec{G}}$. The action ${\varvec{a}}_t$ denotes the possible rigid, affine, or non-rigid transformation to the vertices. To efficiently handle the high dimensional continuous action space, we use *actor* networks $\mu _a ({\varvec{o}}_t;\theta _a)$ to map the observation ${\varvec{o}}_t$ in each step *t*, into a finite set of possible actions, ${\mathcal {A}}_t$, given hyperparameters $\theta _a$. Subsequently, *value* networks $\mu _v({\varvec{o}}_t,{\varvec{a}}_t;\theta _v)$ with $\theta _v$ denoting the hyperparameters are used to predict the associated *Q*-values, and thereby select the policy $\pi _t$ accordingly. therefore, we could formulate our *Q* value function as shown in Eq. (5) and use reinforcement learning to solve this optimization problem.

**Figure 1:**
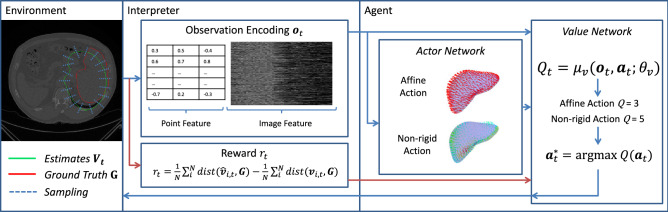
Illustration of the general MDP pipeline using segmentation as the application of interest. The red arrow is for the training phase only and the blue arrows are shared between the training and testing phase. In this example, we segment and register a liver 3D surface mesh to the CT image. The scalar *N* denotes the number of vertices, the point feature comprises the normalized point coordinates, and the image feature shows the gray values sampled along the normal directions at each point. The actor network estimates the affine and non-rigid action given the observation, and the value network calculates the associated *Q*-values in each case. In this case, the *Q*-value for non-rigid action is bigger. Therefore, the non-rigid action will be performed.

An illustration of the overall pipeline is shown in Fig. 1. For current *estimates* ${\varvec{V}}_t$ at the time step *t*, we extract the observation encoding ${\varvec{o}}_t$. Using this observation ${\varvec{o}}_t$, the actor networks predict a finite set of actions ${\varvec{a}}_t \in {{\mathcal {A}}_t}$. Using both the observation ${\varvec{o}}_t$ and action set ${\varvec{a}}_t \in {{\mathcal {A}}_t}$, the value networks predict the associated *Q* value and determine the policy. Each step is explained in detail in the subsequent sections.

5 $$\begin{aligned} Q^*({\varvec{o}},{\varvec{a}}) = \mu _v({\varvec{o}}_t, \mu _a({\varvec{o}}_t ;\theta _a); \theta _v) = \max _\pi {\mathbb {E}}\left[ \sum _{\tau =t}^{\infty }\gamma ^{\tau -t} r_t|{\varvec{o}} = {\varvec{o}}_t, {\varvec{a}}_t = \mu _a({\varvec{o}}_t ;\theta _a), \pi \right] \end{aligned}$$

### Observation encoding

The observation encoding ${\varvec{o}}_t$ is generated by sampling the underlying environment based on current *estimates* ${\varvec{V}}_t$. Depending on the target application, an optional pre-processing step (apart from data normalization) may be applied to the underlying volume. This encoding also depends on whether we are working with a surface mesh or a point cloud. In the case of 3D surface meshes, we calculate the normal vector ${\varvec{n}}_{i,t}$ for each vertex ${\varvec{v}}_{i,t} \in {\varvec{V}}_t$. We use an equidistant sampling pattern along the normal vector, and extract pixel values from the environment. When working with unstructured 3D point clouds, we sample a sub-volume centered on each point ${\varvec{v}}_{i,t} \in {\varvec{V}}_t$, and extract the associated voxel values from the environment. In both cases, the sampled values are vectorized and normalized to zero mean, unit standard deviation, to generate the final image feature. To deal with various voxel spacings in different data, the sampling is done using mm as unit. We also include the spatial positions of the vertices of the *estimate* ${\varvec{V}}_t$ (and the corresponding normal vector ${\varvec{n}}_{i,t}$ if available) to describe the distribution of the mesh vertices. They are also normalized into unit sphere, and describe the point feature. The observation encoding ${\varvec{o}}_t$ is a concatenation of these image features and point features, respectively. An example of observation encoding is shown in Fig. 1.

### Actor networks

The *actor* network maps the observation to a possible action set ${\mathcal {A}}_t$. We define the action set ${\mathcal {A}}_t$ as the union of a rigid translation action ${\varvec{t}}_t = [t_x,t_y,t_z]^T$, scaling action ${\varvec{s}}_t = [s_x,s_y,s_z]^T$, and rotation action ${\varvec{R}}_t(r_x,r_y,r_z)$, and a non-rigid deformation ${\varvec{D}}_t = [{\varvec{d}}_1,{\varvec{d}}_2, \cdots {\varvec{d}}_N]^T\in {\mathbb {R}}^{N \times 3}$. Using imitation learning, an associated actor network $\mu _a$ is trained to predict an action ${\varvec{a}}_t$ given a current observation ${\varvec{o}}_t$. In other words, the actor network is trained to imitate the behavior of a demonstrator for a given observation. In this context, the demonstrator provides the optimal rigid or non-rigid action ${\varvec{a}}^*_t \in {{\mathcal {A}}^*_t} = \{ {\varvec{R}}_t^*, {\varvec{t}}_t^*, {\varvec{s}}_t^*, {\varvec{D}}_t^* \}$. In the training phase, the *ground truth* ${\varvec{G}}$, describing the correct vertex positions, is given. Therefore, we can calculate the optimal action ${\varvec{a}}^*_t$ by minimizing Eqs. (6)–(9). The operation $\circ$ denotes element-wise multiplication, and ${\mathcal {N}}({\varvec{v}}_{i,t})$ is the neighbourhood of the vertex ${\varvec{v}}_{i,t}$. In case ${\varvec{v}}_{i,t}$ belongs to a mesh, the ${\mathcal {N}}$ is defined by the adjacent faces of the mesh. When dealing with point clouds, the k-nearest neighbour algorithm is used to determine the neighbourhood ${\mathcal {N}}$. The regularization term in Eq. (6) makes sure that the deformation vector ${\varvec{d}}_{i,t}$ for vertex ${\varvec{v}}_{i,t}$ is similar to the neighboring deformation vector ${\varvec{d}}_{j,t}$ for vertex ${\varvec{v}}_{j,t} \in {\mathcal {N}}({\varvec{v}}_{i,t})$. This ensures the smoothness of the overall deformation.

6 $$\begin{aligned} {\varvec{D}}^*&= \arg \min _{{\varvec{D}}} \biggr ( {\text {dist}}( {\varvec{D}} + {\varvec{V}}_t, {\varvec{G}} ) + \lambda \sum _i^N\sum _{j \in {\mathcal {N}}({\varvec{v}}_{i,t})} \Vert {\varvec{d}}_{j,t} - {\varvec{d}}_{i,t} \Vert _2^2 \biggr ) \end{aligned}$$

7 $$\begin{aligned} {\varvec{R}}^*&= \arg \min _{{\varvec{R}}} \frac{1}{N}\sum _i^N {\text {dist}}\left( {\varvec{R}}{\varvec{v}}_{i,t}, {\varvec{G}} \right) \end{aligned}$$

8 $$\begin{aligned} {\varvec{s}}^*&= \arg \min _{{\varvec{s}}} \frac{1}{N}\sum _i^N {\text {dist}}\left( {\varvec{v}}_{i,t} \circ {\varvec{s}}, {\varvec{G}} \right) \end{aligned}$$

9 $$\begin{aligned} {\varvec{t}}^*&= \arg \min _{{\varvec{t}}} \frac{1}{N}\sum _i^N {\text {dist}}\left( {\varvec{t}} + {\varvec{v}}_{i,t}, {\varvec{G}} \right) \end{aligned}$$

Due to the fact that the observation ${\varvec{o}}_t$ encodes only the neighbourhood, we suffer from a partial observation effect. To facilitate training of the *actor*, we normalize the output of the demonstrator. The action ${\varvec{a}}^*_t \in {\mathcal {A}}^*_t$ is either normalized to a unit sphere (${\varvec{D}}^*$ and ${\varvec{t}}^*$), or to a specific range (${\varvec{s}}^*$ and ${\varvec{R}}^*$), and denoted as ${{\mathcal {A}}'_t} = \{{\varvec{D}}'_t, {\varvec{t}}'_t, {\varvec{s}}'_t, {\varvec{R}}'_t(r_x, r_y, r_z)\}$. In this way, the *actor* network predicts reasonable actions moving along the same direction as those of the demonstrator. At the same time, the predicted actions do not overstep the range of the observation encodings. Subsequently, we introduced an acceleration term $\beta \in {\mathbb {R}}^+$ during training, and define the target actions ${\varvec{a}}'_{t,\beta } \in {{\mathcal {A}}'_{t,\beta }} = \{\beta \circ {\varvec{D}}'_t, \beta \circ {\varvec{t}}'_t, \beta \circ {\varvec{s}}'_t, {\varvec{R'}}_t(\beta r_x, \beta r_y, \beta r_z)\}$. The action loss function ${\mathcal {L}}_a$ can thus be defined as

10 $$\begin{aligned} {\mathcal {L}}_a = \sum _{{{\varvec{a}}'_{t,\beta }} \in {{\mathcal {A}}'_{t,\beta }}} \Vert {\varvec{a}}'_{t,\beta } - \mathbf {\mu }_a({\mathbf {o}}_t ; \mathbf {\theta }_a) \Vert _2^2 + \lambda _1 \Vert {\mu _a}({\varvec{o}}_t; \theta _a) - \beta \Vert _2^2 \end{aligned}$$

In this equation, the first term denotes the $\text {L}_2$ loss computed using the predicted action, and the second term regularizes the norm of the action to $\beta$. We also introduce an auxiliary loss that computes the distance between the predicted $({\varvec{V}}_t)$ and ground truth $({\varvec{G}})$ vertices as,

11 $$\begin{aligned} {\mathcal {L}}_d = \frac{\lambda _2}{N}\sum _{{\varvec{v}}_{i,t} \in {\varvec{V}}_t} \text {dist}({\varvec{v}}_{i,t}, {\varvec{G}}) \end{aligned}$$

**Figure 2:**
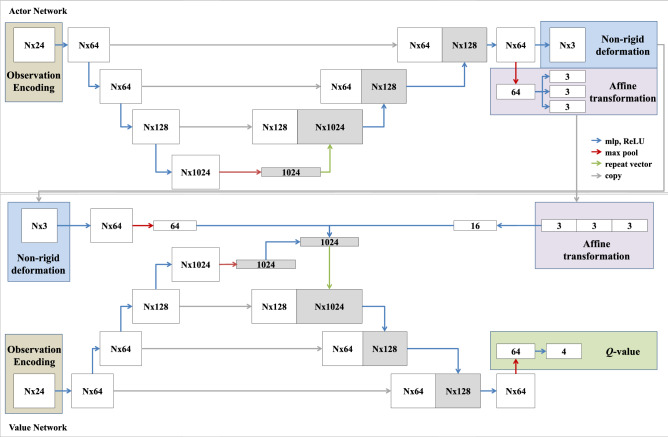
Illustration of the network architecture for the *actor* network (top) and *value* network (bottom), respectively. Feature maps are represented as boxes. The scalar *N* denotes the number of vertices. The dimension of the observation encoding block depends on the number of sampling points and on the presence of the normal vector.

The scalar $\lambda _1$ and $\lambda _2$ are the Lagrangian multiplier. The combined loss evaluated as ${\mathcal {L}}_a^g = {\mathcal {L}}_a + {\mathcal {L}}_d$, was used for training. Furthermore, we adopted a multi-task training strategy for the *actor* network $\mathbf {\mu }_a$, based on the rigid and non-rigid actions to be estimated. This improved network stability during training and reduced the overall computational complexity. The network architecture is depicted in Fig. 2.

### Value network

In order to determine the best action $${\varvec{a}}_t^*$$ in the predicted action set $${\mathcal {A}}_t$$ using the *actor* network $$\mu _a$$, such that the sum of current and expected cumulative future reward is maximized, a $$value $$ network is trained to estimate the *Q*-value for each action $${\varvec{a}}_t$$. To this end, we use the *Q*-learning framework and define the *value* network as $$\mu _v({\varvec{o}}_t,{\varvec{a}}_t;\theta _v)$$, where $$\theta _v$$ represents the associated hyperparameters. The instant reward at step *t* is expressed as

12 $$\begin{aligned} r_t = \frac{1}{N} \sum _i^N{\text {dist}}(\hat{{\varvec{v}}}_{i,t}, {\varvec{G}}) - \frac{1}{N} \sum _i^N{\text {dist}}({\varvec{v}}_{i,t}, {\varvec{G}}) \end{aligned}$$

where $\hat{{\varvec{v}}}_{i,t}$ denotes the current estimate of ${\varvec{v}}_{i,t}$ applying predicted action ${\varvec{a}}_t$. This indicates, the relative reduction of the distance between *estimates* and the *ground truth*. The optimal action-value function $Q^*$ is defined using Eq. (1), and the loss function can be found in Eq. (2). To facilitate training of the *value* network, we used a double deep *Q*-network (DDQN)^34^, where a separate *target network* $\mu _{v}^-({\varvec{o}}_t, {\varvec{a}}_t;\theta ^-)$ is introduced to approximate $Q^*$ as,

13 $$\begin{aligned} Q^* \approx r_t + \gamma \max _{{\varvec{a}}_{t+1}}\mu _{v}^-({\varvec{o}}_{t+1},{\varvec{a}}_{t+1};\theta ^-) \end{aligned}$$

In Eq. (13), $\theta ^-$ represents the hyperparameters of the *target* network. Both networks share the same architecture, but $\theta ^-$ is updated slowly i.e. once every few iterations, while $\theta _v$ is updated every iteration. This strategy contributes to the overall convergence of the network(s), during training.

We determine the boundary condition of the *Q*-value to facilitate training of the *value* and *target* network. The action ${\varvec{a}}_t$ is estimated using the *actor*, and the magnitude of the action is constrained by the normalization and the acceleration factor $\beta$. Accordingly, we approximate the upper bound of the reward function as, $r_t^+ \approx \beta$. Furthermore, we approximate the average action loss as $\bar{{\mathcal {L}}} = \frac{1}{2}{L}_a^g$. This in turn enables us to approximate the lower bound of the reward function, $r_t^- \approx -\sqrt{\bar{{\mathcal {L}}}} > -\beta$. As the reward function is bounded, we determine the boundary condition of the *Q*-value using sequence limit, as expressed in,

14 $$\begin{aligned} \frac{r_t^-}{(1-\gamma )}< Q^*< \min \left( \frac{r_t^+}{(1-\gamma )} \ , \ d_{max}\right) \end{aligned}$$

Here, $d_{max}$, denotes the maximal distance between the *estimates* and the *ground truth*. The value of $d_{max}$ can be calculated according to the data augmentation strategy employed.

Although we have shown in Eq. (14) that the *Q*-value is bounded, it is not practical to set the number of steps $t \rightarrow \infty$, during inference. This is why we employ two termination criteria for the action sequence chosen by the *value* network. First, we introduce $t_{max}$ as the maximal number of steps in a sequence. This defines the maximum run-time of the algorithm. The second criterion is that the *Q* value predicted by the *value* network should be greater than $Q_{min}$. If the predicted value is less than $Q_{min}$, we consider the deformation to be insignificant, and terminate the sequence.

### Data augmentation

We use data augmentation to generate sufficiently rich data for training the *actor* and *value* networks. Data augmentation is applied to both the observation encoding ${\varvec{o}}_t$ and the *estimates* ${\varvec{V}}_t$. For the observation encoding, Gaussian noise was applied to the image features. Data augmentation on the *estimates* ${\varvec{V}}_t$ was performed by deforming the *ground truth* surface meshes ${\varvec{G}}$ used for training. In order to ensure that the deformations of the *ground truth* meshes were realistic, we trained a statistical shape model (SSM) of the target structure, by template-to-sample registration using the coherent point drift (CPD) algorithm. This registration is done iteratively, where in the first iteration, a random sample is chosen to be the template. In each iteration, the template-to-sample registration is performed, and the template is updated by calculating the average model at the end of each iteration. We then deformed the *ground truth* ${\varvec{G}}$ using the modes of variation of the trained SSM, followed by the application of random affine transformations. Finally, each vertex was randomly jittered using small displacements, and subsequently, the mesh was smoothed. Note that the point correspondence imposed by the SSM is applied only to the generated training data. The calculation of the optimal action ${\varvec{a}}^*_t$ of the demonstrator requires no correspondence between the vertices. Therefore, the deformation estimated by the *actor* remains independent of point correspondences.

### Training

The first step towards training the proposed framework is to train the *actor* network. To this end, the Dataset Aggregation (DAGGER) algorithm^35^ was employed to efficiently sample the high dimensional training data space for a sequential process. The pseudo code for the DAGGER algorithm is presented in Alg 1. In the first iteration of the DAGGER algorithm, the *actor* is trained using the training data ${\mathcal {D}}$ generated by the demonstrator. Subsequently, new data ${\mathcal {D}}_\pi$ was gathered by applying the *actor*, and extracting the observation and optimal action set generated using the demonstrator. These data ${\mathcal {D}}_\pi$ are aggregated with the training dataset ${\mathcal {D}}$, for the next iteration of training.

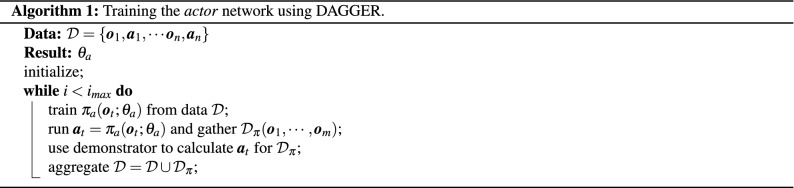



This training scheme is directly applicable to the non-rigid action. When training the network in a multi-tasking fashion for the rigid actions, we need a criterion to select the action ${\varvec{a}}_t$ to further gather training data ${\mathcal {D}}_\pi$. Here, we select randomly one of the predicted translation, rotation and scale as ${\varvec{a}}_t$.

Following this initial training step, the *actor* and *value* networks are jointly trained using the Deep Deterministic Policy Gradients (DDPG) approach^28^. The pesudo code of the DDPG algorithm is presented in Alg. 2. Here, the *value* network (referred to as the critic-network in^28^) is trained using the DDQN algorithm with an $\epsilon$-greedy action selection scheme. The gradient of the *actor* can be calculated using the chain rule to compute the derivatives of the associated hyperparameters, and update them accordingly. As our *actor* network is pre-trained using imitation learning, we update our *actor* at a much slower rate than the *value* network.

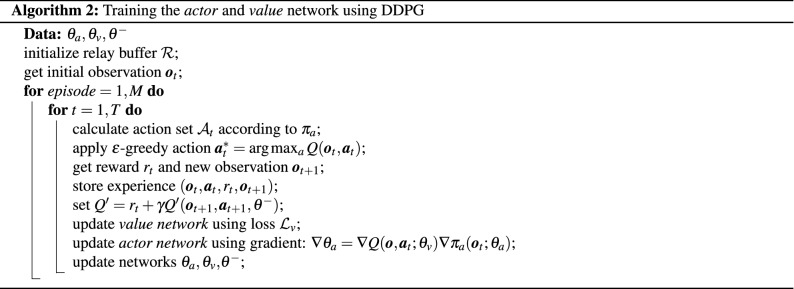



### Network architecture

The proposed architecture for the *actor* and *value* network is shown in Fig. 2. Both networks have shared layers designed to extract high-level features from the encoded observations. This shared-layer architecture was inspired by the PointNet^36^, where a dense layer was used to extract vertex-wise local features. These features are merged using a pooling layer, enabling inter-vertex global features to be learned. The extracted global features are permutation invariant, and therefore do not pose any restrictions on the structure of the neighbourhood. The global feature is repeated and aggregated with the local vertex-wise features using skip connections, as done in the U-Net. The output layer for the *actor* network, predicts the non-rigid action as a deformation vector for each vertex, and the affine action in the form of an affine transformation with nine degrees of freedom. Consequently, the output layer is constructed to fit the outputs of the *actor*, where, for the rigid action, an additional pooling layer is used to encode each action (i.e. rotation, translation and scaling) as a vector. For the *value* network, the predicted actions are considered as additional input and concatenated with the global inter-vertex features extracted using the shared network architecture. The outputs of the *value* network are the *Q*-values estimated for the associated actions.

## Results

We evaluated our method for two different tasks, namely, segmentation and registration. For the former, the performance of our approach was evaluated for mono- and multi-modal liver segmentation and compared to a state-of-the art approach, namely the 3D U-Net. In addition, the registration performance of our approach was evaluated for the challenging task of intra-operative brain shift compensation based on multi-modal images. Furthermore, we investigated the effect of the acceleration factor $\beta$ used when training the *value* network, assessed the convergence of the algorithm, and analyzed its computational complexity. All computations during both training and evaluation were performed on the same machine using an NVIDIA GTX 1070 GPU.

### Mono-modality image segmentation

In the first set of experiments, evaluating mono-modal segmentation performance, we trained our framework using the Visceral Anatomy3^37^ CT datasets, comprising 20 CT volumes with ground truth segmentations for the liver. The dataset was partitioned into training, validation, and test sets, using a split of 16 : 2 : 2. The 3D U-Net was used as a baseline for comparison with our approach. To this end, it was trained and evaluated using the same samples. All volumes were normalized such that their intensity values are within the range of [0, 1]. Due to clinical runtime requirements and hardware restrictions imposed by the target application, the input to the 3D U-Net had to be resampled. To this end, we cropped the original volumes to ROIs centered around the liver, and resampled the ROIs to a resolution of $128\times 128\times 128$.

A DICE loss function, as proposed in^15^, was used to train the 3D U-Net. In contrast, for the proposed method we could use the volumes in their original resolution and no cropping was performed. As described above, we augmented the data when training the *actor* and *value* networks. This was done by first training an SSM of the liver using each training sample (via template-to-sample registration using CPD). Subsequently, the modes of the SSM were used to generate realistic deformations of the training samples for augmentation. We sampled the image feature along the normal vector of each vertex using 1 mm equal distance over 61 points to extract the gray values. During testing, we placed the template, reshaped using random modes of variation, at different positions in the volumes. Displacements in the rage of $[-40,40]$ mm (i.e. approximately half the size of the liver ROI) in each direction were used. We evaluated both methods by performing a ten-fold cross-validation, using the Anatomy3 CT dataset. In addition, we tested them on unseen samples from the Visceral Silver-Copus CT dataset^37^. The DICE coefficient was used as the metric to evaluate the performance. When comparing results, we state the average (AVG) ± standard deviation (STD) of this metric. The results for both approaches over test samples from the cross-validation experiments, and the unseen samples from the Visceral Silver-Copus dataset, are summarized in Table 1. They show that the proposed method managed to outperform the 3D U-Net despite comprising significantly fewer parameters (2.7 million as opposed to 16.1 million for the 3D U-Net). Furthermore, the training of the 3D U-Net took roughly 72 h while the proposed method was trained approximately for 12 h.

**Table 1:** Mono-modal liver segmentation accuracy, trainable parameters (in millions), and run-time comparison for the Anatomy3 and SilverCopus datasets.

Dataset	No.	Algorithm	Dice	Parameters	Runtime
			[%]	[M]	[s]
			AVG ± STD		
Anatomy3	20	Our method	**90**.**8** ± **0**.**7**	2.7	1
CT		3D U-Net	90.5 ± 3.6	16.1	60
SilverCopus	51	Our method	**89**.**9** ± **0**.**6**	2.7	1
CT		3D U-Net	89.1 ± 3.9	16.1	60

The number of parameters for the proposed methods is the sum of *actor* and *value* networks.

### Multi-modality segmentation

In the multi-modality segmentation task, we evaluated the proposed method on two clinically relevant scenarios. First, we used the models trained in the previous set of experiments (refer to “Mono-modality image segmentation” section) for liver segmentation in single-energy CT volumes, and applied them to volumes acquired at different clinical centers, where different scan protocols were followed to acquire the CT data. This included contrast enhanced CT (CECT) from Visceral Anatomy3, single energy CT from 3D-IRCADb-01, and dual energy CT (DECT) data collected by our clinical partner. For all datasets, we only normalized the intensity as introduced in section 3.1. No further pre-processing was used. To evaluate the robustness of the algorithm, we did not use transfer-learning (i.e. fine-tune the networks) at this stage. We evaluated for each CT volume in each dataset all ten trained models from ten-fold cross-validation performed in “Mono-modality image segmentation” section. This was done for both proposed method as well as for the 3D U-Net. Finally, we documented the AVG ± STD DICE score. The segmentation results achieved by the proposed approach and 3D U-Net, for all three types of CT datasets tested, are summarized in Table 2. The results show that the DICE score of the proposed method was consistently and significantly higher than what could be achieved with the 3D U-Net.

**Table 2:** Multi-modal liver segmentation accuracy using pre-trained CT model for Anatomy3 CECT, 3DIrcad and DECT datasets acquired with different scan protocols, and cross-validation on CHAOS CT-MR dataset.

Dataset	No.	Our Method	3D U-Net
		DICE [%]	DICE [%]
		AVG ± STD	AVG ± STD
Anatomy3 CECT	20	**82**.**6** ± **7**.**6**	72.4 ± 9.9
3DIrcad	20	**82**.**3** ± **9**.**0**	59.4 ± 11.1
Dual Energy CT	45	**86**.**5** ± **3**.**5**	60.5 ± 10.5

The accuracy of CHAOS CT-MR dataset is further broken down into individual modalities.

**Figure 3:**
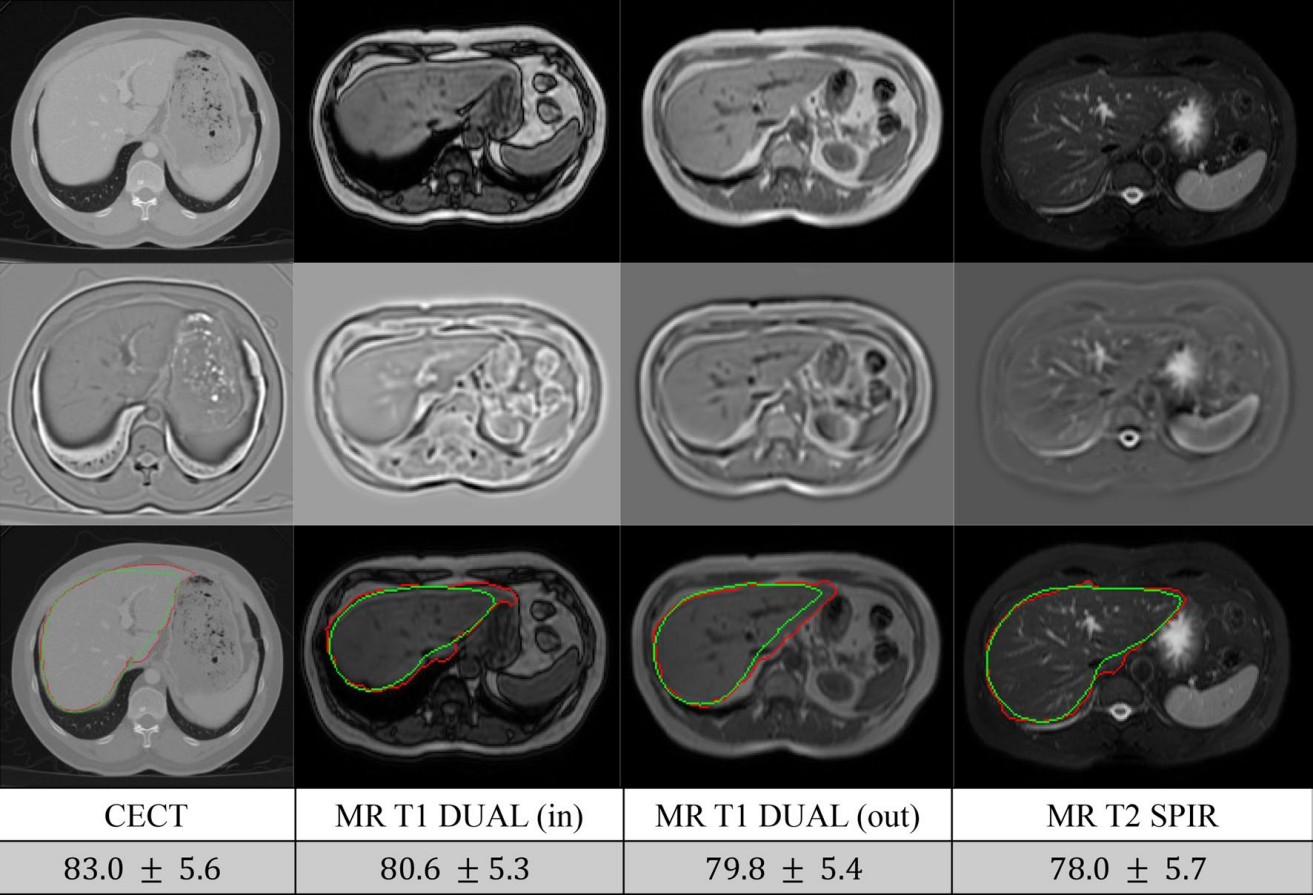
An example of the CHAOS multi-modal segmentation dataset. The top row shows the original images (CECT, MRI T1 in-phase, MRI T1 out-phase, MRI T2-SPIR). The middle row shows associated pre-processed images using the LoG filter. The bottom row displays the original images together with the segmentation estimates (green) and ground truth (red).

Second, we evaluated our model using the multi-modal CHAOS challenge dataset. This dataset comprises a total of 40 contrast enhanced CT (CECT) volumes, 40 T1 in phase MRI volumes, 40 T1 out phase MRI volumes, and 40 T1 SPectral Attenuated Inversion Recovery (SPAIR) MRI volumes of the abdomen. We trained our network using all modalities simultaneously, without incorporating modality-specific prior knowledge. This is a challenging task as the appearance and spatial resolution of the data vary significantly. To deal with the large differences in appearance between MRI and CT, we pre-processed all volumes using a Laplacian of Gaussian filter (LoG). As depicted in Fig. 3, the LoG filtered images are relatively similar in appearance, unlike the original images. Each volume was then normalized to unit variance and zero mean. The rest of the pipeline of the proposed method remained the same, despite the differences in spatial resolution of the images, as all steps in the proposed method were in the world coordinate system using mm as unit instead of the number of voxels. We trained the networks using this dataset, then evaluated the performance by applying a ten-fold cross-validation. In the ten-fold cross-validation, we formed 40 sets, each comprising a volume of each modality. In other words, each set included one CECT volume, one T1 in phase volume, one T1 out phase volume, and one T1 SPAIR volume. The ratio between training, validation and testing for these 40 sets was 32:4:4. As shown in Fig. 3, using the proposed method, a single trained network can segment input volumes of different modalities. The segmentation performance is comparable for all different modalities despite the difference in appearances and spatial resolutions.

### Registration

**Figure 4:**
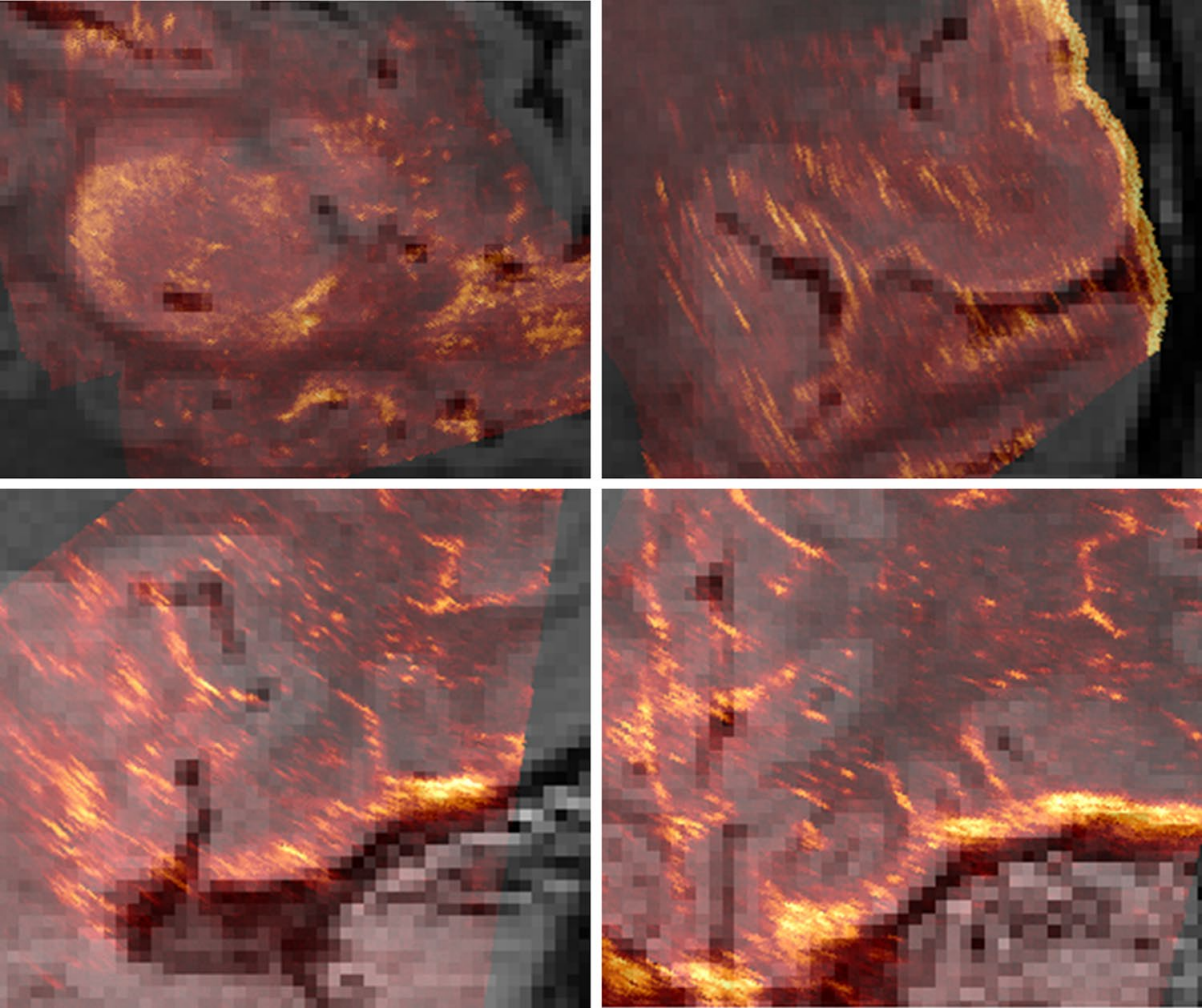
Two examples of MR Images (grey scale) combined with registered iUS volume overlays (yellow-red image) using the proposed method. Each row represent a dataset. Left: sagittal view, right: coronal view.

For the registration application, we picked brain-shift compensation^32^. The network was trained and cross-validated using the Correction of Brainshift with Intra-Operative Ultrasound (CuRIOUS) MICCAI challenge 2018 training dataset. In total 20 datasets were used. They were partitioned into training, validation, and testing, using a split of 16:2:2. In this setting, we encoded the observation by sampling a $7 \times 7 \times 7$ sub-volume centered at each landmark without any preprocessing step and relied on the *actor* to estimate associated deformation vectors. Since the volume was registered to the pre-operative data via an optical tracker on the ultrasound probe during the intervention^38^, we only used the non-rigid action in this setting. Therefore, we employed only the DAGGER to train the *actor* but no *value* network was involved. The *actor* network architecture was the same as illustrated in Fig. 2 with small variations in the input layer due to difference in observation encoding. We further changed the base number of feature kernels from 64 to 32. As the number of landmarks varied in the data, we introduced a constant, *N*, chosen such that the maximum number of landmarks could be accommodated. In case of data with fewer landmarks, random landmarks were copied in the input. This random copying had no impact on the network, both, during training and testing phase, as the vertex-wise deformation predicted by the network and the max pooling value remains the same in case of duplicated values. To evaluate the performance of the proposed method, we used ten-fold cross-validation and applied the trained networks to the landmarks in the test set. Then we calculated the mean target registration error in mm between estimated and ground truth position of the all landmarks. We used the NiftigReg method^39^ as baseline for the registration. The registration result is shown in Table 3, and examples of the MRI overlaid with registered iUS frames are depicted in Fig. 4. As we can see, the proposed method compensated the deformation introduced by brain-shift well.

**Table 3:** Evaluation of the mean distance between landmarks in MRI and ultrasound before and after correction.

ID	Landmarks	Mean distance (range)	Mean distance (range)
	Number	Initial in mm	Corrected in mm
1	15	1.82 (0.56–3.84)	0.88 (0.25–1.39)
2	15	5.68 (3.43–8.99)	1.01 (0.42–2.32)
3	15	9.58 (8.57–10.34)	1.10 (0.30–4.57)
4	15	2.99 (1.61–4.55)	0.89 (0.25–1.58)
5	15	12.02 (10.08–14.18)	1.78 (0.66–5.05)
6	15	3.27 (2.27–4.26)	0.72 (0.27–1.26)
7	15	1.82 (0.22–3.63)	0.86 (1.72–0.28)
8	15	2.63 (1.00–4.15)	1.45 (0.73–2.40)
12	16	19.68 (18.53–21.30)	2.27 (1.17–4.31)
13	15	4.57 (2.73–7.52)	0.96 (0.31–1.44)
14	15	3.03 (1.99–4.43)	0.87 (0.31–1.92)
15	15	3.32 (1.15–5.90)	0.69 (0.23–1.17)
16	15	3.39 (1.68–4.47)	0.83 (0.34–1.96)
17	16	6.39 (4.46–7.83)	0.96 (0.31–1.61)
18	16	3.56 (1.44–5.47)	0.89 (0.33–1.33)
19	16	3.28 (1.30–5.42)	1.26 (0.41–1.74)
21	16	4.55 (3.44–6.17)	0.85 (0.26–1.33)
23	15	7.01 (5.26–8.26)	1.08 (0.28–3.40)
24	16	1.10 (0.45–2.04)	1.61 (0.52–2.84)
25	15	10.06 (7.10–15.12)	1.76 (0.62–1.76)
26	16	2.83 (1.60–4.40)	0.93 (0.47–1.44)
27	16	5.76 (4.84–7.14)	2.88 (0.79–5.45)
Proposed		5.37 ± 4.27	1.21 ± 0.55
NiftiyReg^39^		5.37 ± 4.27	2.90 ± 3.59

### Computational complexity

To analyze the computational complexity, we consider the trained networks as ${\mathcal {O}}(1)$ as its parameters are fixed. Our method has a complexity of ${\mathcal {O}}(n \cdot m)$ for both *actor* and *value* network, where *n* denotes the number of vertices and *m* denotes the number of features, independent of the 3D volume resolution. In contrast, 3D U-Net and its variations/extensions have a complexity of ${\mathcal {O}}(h \cdot w \cdot d)$, where *h* denotes the height, *w* denotes the width and *d* denotes the depth of the volume all in voxels (3D matrix size). Therefore, for an average case with multiple slices (100–300 slices for an abdomen CT scan and a in-plane matrix size of 512 $\times$ 512), our method is less computational demanding. In terms of time complexity, the processing of an input mesh with 1*K* vertices, the *actor* and *value* network require approximately 1 GFLOP regardless of the volume size. At the same time, the base-line 3D U-Net for an input volume size of $128\times 128\times 128$ requires 70 GFLOPs.

### Speed-up factor $\beta$

**Figure 5:**
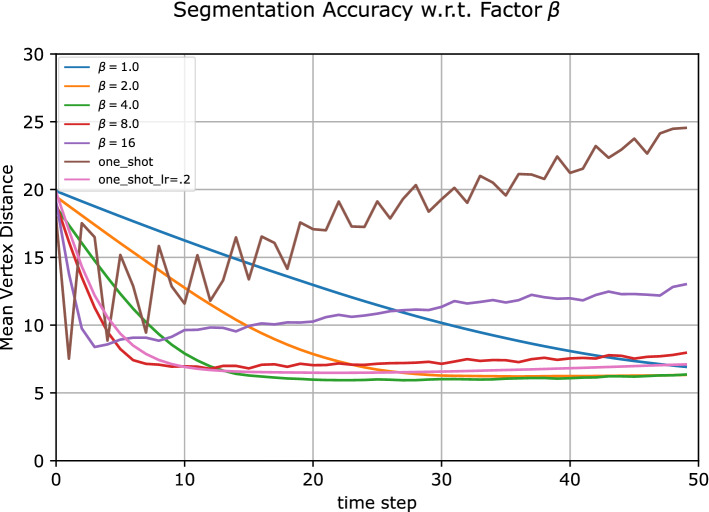
The impact of different acceleration factors $\beta$ w.r.t to the mean vertex distance of the segmentation. We also compare results obtained using one-shot prediction (one_shot), vs one-shot with learning rate (one_shot_lr).

We analyzed the actor network w.r.t. the key variable, the speed-up factor $\beta$. We chose the liver segmentation as our target application and investigated the impact of this parameter on the Anatomy3 data. To facilitate the investigation, we chose the *actor* network for non-rigid registration to evaluate the impact on performance and general convergence using different $\beta$ values. We trained the *actor* network using different speed-up factors $\beta$ and evaluated it on one set of test data to investigate its overall impact to the segmentation performance and general convergence. For a more comprehensive comparison, we also included the one-shot estimation and one-shot with learning rate. The *actor* network in one-shot approach learns directly the optimal action while one-shot with learning rate learns the optimal action multiplied with the learning rate. To study the effect of different values for $\beta$, we evaluated multiple trained *actor* networks associated with different $\beta$ values and ploted the mean vertex distance of the test set over 50 time steps. As shown in Fig. 5, the value of $\beta$ was directly correlated to the convergence speed of the algorithm over the time step *t*.

## Discussion and outlook

Our results suggest that the proposed method provides good accuracy and robustness for segmentation and registration applications in single and multi-modal datasets. While the accuracy for each specific scenario could be further improved by using specific pre-processing or post-processing methods, we refrained from this, as we wanted to keep our approach general and flexible. In addition, different data splits and other baseline methods could be used to find out how the use of other training datasets impacts the proposed method relative to others. This is, however, part of future work as the number of suitable training datasets currently available to us is limited.

Since the proposed method samples the observation encoding in the volume using absolute dimensions (mm) instead of voxels as base unit, the algorithm can be readily applied to datasets having different voxel sizes. In particular, no resampling during both training and application phase is required. This property is especially useful for multi-modal applications where spatial resolution may vary across different imaging modalities. Also note that, our method requires a proper computation of an affine transformation matrix between the Right, Anterior, Superior (RAS+) coordinate system and the voxel coordinate system. In case of clinical datasets, this matrix can be retrieved or computed using Neuroimaging Informatics Technology Initiative (NII) or DICOM header files. However, in some segmentation challenge dataset, this relevant information is missing or misleading. In this case, the proposed method will fail, while methods only relying on voxel data might still work.

In the multi-modal segmentation application we have found that the proposed method still achieves good performance on CECT and DECT datasets, even when trained on only one type of CT data. On the other hand, the 3D U-Net struggled with different modalities. One plausible explanation is that the proposed method does not learn directly from the raw intensity values, whereas the 3D U-Net does. This makes the change of the intensity values found within the liver, due to contrast agent and different energy levels, detrimental to the performance of the 3D U-Net. Such changes have less impact on our method, as we learn actions leading to a correct solution. This robustness was confirmed during the experiments involving the CHAOS dataset, where LoG pre-processing was used.

We found that large values of $\beta$ yielded faster convergence but worse accuracy, while small values of $\beta$ resulted in slower convergence but higher precision. As expected, the value of $\beta$ is a trade-off between speed and the accuracy of the network. Using one-shot, the network diverged after the first iteration. By introducing a learning rate, we can clearly see that the algorithm stabilized over time.

The introduction of the action normalization and the speedup factor $\beta$ plays an important role in the training process. Although it has been shown that the registration can be done using CNN in a one-step approach^40^, this may not be the optimal approach. If we consider image registration as a sequential energy minimization process, the one-step approach corresponds to an algorithm finding the solution using a very large learning rate. Such an algorithm may, however, not converge to a minimum, as shown in Fig. 5. In addition, it may not generalize well when applied to unseen datasets^41^. By normalizing the action, we are effectively setting the step size of the algorithm to a fixed number. This strategy outperformed a fixed learning rate due to a combination of feature encoding and the optimization of the loss function. In consecutive steps, the feature encoding remains rather similar, but the magnitude of the action is constantly changing (due to the learning rate). As a consequence, the L2 loss function in this ill-posed problem is effectively trying to find an average magnitude of the actions.

**Figure 6:**
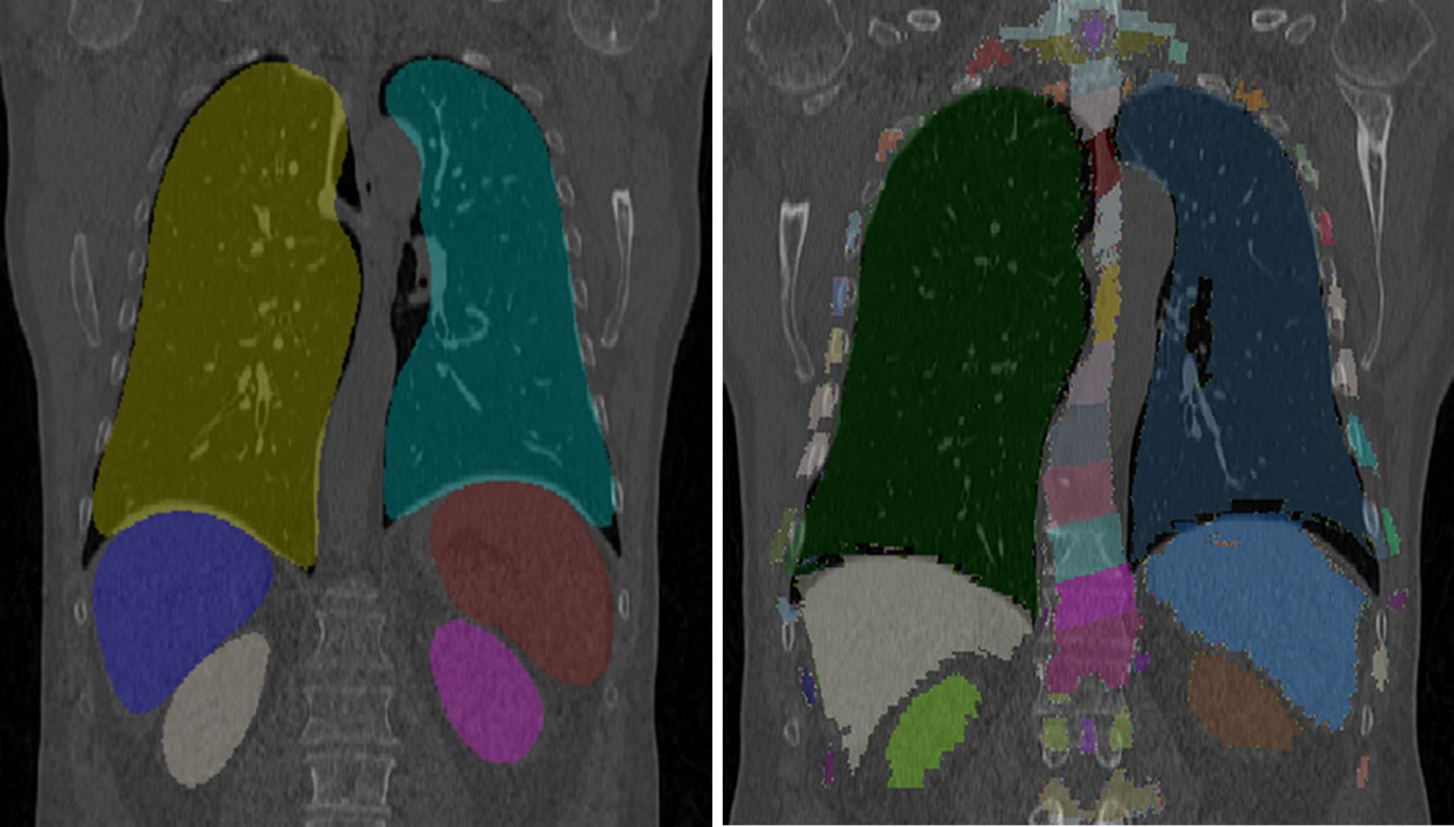
An example of multi-organ segmentation of a CT dataset (left) and warped Visible Human Phantom segmentation overlay (right).

As all point correspondences are modeled only implicitly, another potential application of our method is to register computational phantoms, e.g., the Visible Human (VisHum)^42^ to CT volumes. This warping propagates the dense segmentation in the phantom to the CT volume and can facilitate new clinical application, e.g., patient X-ray dose estimation. In such a case, we train our agent e.g., on lungs, liver, spleen and kidneys. To register the Visible Human to a CT dataset, we can use the segmentation of the Visible Human as initialization and then estimate associated deformation vectors of the organs. Afterwards, a dense segmentation field can be interpolated using these sparse deformation vectors via B-spline interpolation. Using the deformation field, we finally warp the Visible Human to an arbitrary CT volume. An example of this application is shown in Fig. 6. As we can see, the visual propagation of the dense segmentation is quite accurate, and our approach can accomplish this in seconds.

In sum, due to its flexibility, robustness, and relatively low computational complexity, the proposed method provides a promising multi-purpose segmentation and registration framework, particular in the context of image-guided interventions. In subsequent work, more clinical applications will being investigated
